# The Role of Obesity in Frailty Incidence: The San Antonio Longitudinal Study of Aging

**DOI:** 10.1093/geroni/igab046.3026

**Published:** 2021-12-17

**Authors:** Tiffany Cortes, Chen-pin Wang, Helen Hazuda, Sara Espinoza

**Affiliations:** 1 University of Texas HSC at San Antonio, San Antonio, Texas, United States; 2 UT Health San Antonio, UT Health San Antonio, Texas, United States; 3 University of Texas Health Science Center San Antonio, San Antonio, Texas, United States

## Abstract

Background: Although initially conceptualized as a wasting syndrome, obesity has been associated with frailty in prior studies. The goal of this study was to examine the associations of obesity and waist circumference with frailty and determine whether they predict incident frailty in an ethnically diverse population of older Mexican Americans (MAs) and European Americans (EAs). Methods: 749 MA and EA community-dwelling older adults (65+) participated in the baseline examination of the San Antonio Longitudinal Study of Aging (SALSA), and 474 participants completed the first follow up approximately 6 years later. Frailty was classified using Fried criteria. Baseline characteristics, including body mass index (BMI) and waist circumference (WC) were summarized by frailty category (non-frail, pre-frail, frail) using ANOVA. The odds of becoming frail at follow-up by baseline BMI and WC were estimated using separate logistic regression models, adjusting for age, sex, ethnicity, diabetes, comorbidity (presence of ≥2 chronic diseases not including diabetes), baseline frailty score, and follow-up time. Results: At baseline, participants were 69 ±3 years old, 61% female, and 50% MA. BMI and WC increased with increasing frailty category (p <0.01 for both). BMI was a significant predictor of incident frailty (OR=1.08, 95% confidence interval [CI]: 1.02-1.14, p=0.011). WC also predicted frailty (OR=1.03, 95% CI: 1.01-1.05, p =0.017). Conclusion: These results demonstrate that BMI and WC are significant predictors of frailty. Interventions which target obesity may reduce the incidence of frailty; however, more research in this area is needed.

